# Thermal–Electrical Fusion for Real-Time Condition Monitoring of IGBT Modules in Transportation Systems

**DOI:** 10.3390/mi17020154

**Published:** 2026-01-25

**Authors:** Man Cui, Yun Liu, Zhen Hu, Tao Shi

**Affiliations:** 1 School of Integrated Circuits and Electronics, Beijing Institute of Technology, Beijing 100081, China; 7520210140@bit.edu.cn; 2 College of Artificial Intelligence, Nanjing University of Posts and Telecommunications, Nanjing 210023, China; liuyun@njupt.edu.cn

**Keywords:** IGBTs, condition monitoring, thermal gradient analysis, rail traction converters

## Abstract

The operational reliability of Insulated Gate Bipolar Transistor (IGBT) modules in demanding transportation applications, such as traction systems, is critically challenged by solder layer and bond wire failures under cyclic thermal stress. To address this, this paper proposes a novel health monitoring framework that innovatively synergizes micro-scale spatial thermal analysis with microsecond electrical dynamics inversion. The method requires only non-invasive temperature measurements on the module baseplate and utilizes standard electrical signals (load current, duty cycle, switching frequency, DC-link voltage) readily available from the converter’s controller, enabling simultaneous diagnosis without dedicated voltage or high-bandwidth current sensors. First, a non-invasive assessment of solder layer fatigue is achieved by correlating the normalized thermal gradient ($\nabla T_{P}$) on the baseplate with the underlying thermal impedance ($Z_{\mathrm{JC}}$). Second, for bond wire aging, a cost-effective inversion algorithm estimates the on-state voltage ($V_{\mathrm{ce},\mathrm{on}}$) by calculating the total power loss from temperature, isolating the conduction loss ($P_{\mathrm{cond}}$) with the aid of a Foster-model-based junction temperature ($T_{J}$) estimate, and finally computing $V_{\mathrm{ce},\mathrm{on}}$ at a unique current inflection point ($I_{C,\inf}$) to nullify $T_{J}$ dependency. Third, the health states from both failure modes are fused for comprehensive condition evaluation. Experimental validation confirms the method’s accuracy in tracking both degradation modes. This work provides a practical and economical solution for online IGBT condition monitoring, enhancing the predictive maintenance and operational safety of transportation electrification systems.

## 1. Introduction

Insulated Gate Bipolar Transistors (IGBTs) serve as the critical core components for high-voltage, high-power energy conversion, whose reliability fundamentally determines the depth and breadth of modern transportation electrification. In key sectors such as rail transit and electric vehicles, IGBT modules act as the “heart” of traction converters and auxiliary power supply systems, responsible for precise power regulation and energy distribution. However, the operational environment for transportation equipment is extremely complex; traction systems endure sustained high-power loads, severe mechanical vibrations, and wide ambient temperature fluctuations. This harsh operational regime induces significant cyclic thermal stress within IGBT modules, leading to primary failure modes such as bond wire interfacial separation and solder layer delamination. Statistical analyses indicate that approximately 34% of traction system failures can be traced back to power semiconductor devices, with IGBT failure being a predominant cause [1,2,3]. Consequently, developing real-time, accurate online health monitoring technologies for IGBTs is a central challenge for achieving high reliability and predictive maintenance in transportation assets. It also represents a key research focus within the current frontier of digital-intelligent trains and smart operations and maintenance.

Addressing this challenge, existing research on IGBT health monitoring primarily revolves around two categories of methods: thermal-sensitive and electrical-sensitive parameters. Thermal approaches assess aging by monitoring junction temperature dynamics or thermal impedance variations. For instance, the chip-to-baseplate thermal impedance is a key indicator for solder layer aging [4,5,6,7]. However, accurate junction temperature measurement is inherently affected by device aging, while methods like thermal differentials are strongly dependent on operational parameters, requiring the construction of extensive power loss databases, which complicates engineering implementation [8,9]. Electrical methods diagnose degradation by monitoring electrical parameters sensitive to aging, such as the collector-emitter on-state voltage ($V_{\mathrm{ce},\mathrm{on}}$) and gate peak current ($I_{\mathrm{gpeak}}$) [10,11,12,13,14]. While $V_{\mathrm{ce},\mathrm{on}}$ is a classic indicator for bond wire degradation, its minute variations (on the millivolt scale) are easily masked by load current fluctuations and junction temperature drift. This necessitates high-precision sampling circuits, which not only increase system cost and complexity but also face significant challenges in the high-electromagnetic-interference environment of transportation electrical systems [15,16,17,18].

It is noteworthy that the transportation sector imposes more specific and urgent demands on IGBT monitoring technologies. On one hand, national research priorities aim to develop compact, low-intrusive online monitoring solutions to enhance overall traction system safety. On the other hand, as the power ratings of equipment like high-speed trains continue to increase, the thermal management pressure on IGBT modules intensifies. Any localized increase in thermal impedance due to aging can potentially trigger thermal runaway, jeopardizing operational safety. Furthermore, the industry has begun formulating relevant standards to regulate the selection and inspection of IGBTs for rail transit vehicle traction converters, further highlighting the need for reliable, standardized health assessment tools. Therefore, exploring a monitoring methodology that minimizes additional sensing circuitry (particularly high-precision analog voltage measurement), can adapt to harsh on-site environments, and can simultaneously diagnose multiple failure modes holds significant value. The proposed method achieves this by using non-invasive temperature as the primary sensing input and leveraging existing digital controller signals ($I_{C}$, *D*, $f_{\mathrm{sw}}$, $V_{\mathrm{DC}}$) for parameter inversion, thereby avoiding costly and interference-prone dedicated sensor loops.

Recent research reveals that failure in IGBT modules significantly alters their internal heat flow paths and the spatial temperature distribution at the sub-millimeter level across the package surface, offering a new perspective for diagnosis through thermal signatures. Inspired by this, this study proposes a coordinated IGBT health monitoring framework for transportation applications, based on the fusion of micro-scale thermal distribution characteristics and inverse calculation of electrical parameters. The method innovatively combines baseplate multi-point temperature gradient analysis with on-state loss inverse computation, aiming to achieve simultaneous, real-time identification of the two primary failure mechanisms—solder layer fatigue and bond wire aging—using primarily temperature sensing information. The core contributions of this framework are: (1) establishing a solder layer aging monitoring model based on the micro-scale baseplate spatial temperature gradient ($\nabla T_{P}$), enabling non-intrusive diagnosis through a correlated database between $\nabla T_{P}$ and thermal impedance ($Z_{\mathrm{JC}}$); (2) proposing a method to calculate $V_{\mathrm{ce},\mathrm{on}}$ based on inverse calculation of total loss and switching loss. By utilizing a characteristic operating point (where $V_{\mathrm{ce},\mathrm{on}}$ is insensitive to junction temperature), it eliminates the dependency on extensive parameter lookup tables, significantly reducing the cost and complexity of bond wire monitoring. The methodology presented in this study offers a promising solution to meet the pressing need for highly reliable and lightweight monitoring of power devices in transportation equipment.

The remainder of this paper is organized as follows. Section 2 provides a detailed exposition of the proposed spatial multi-parameter thermal inversion methodology for IGBT health diagnosis. Section 3 details the implementation architecture of the proposed framework. Section 4 presents experimental validation through empirical case studies. Section 5 concludes the paper.

## 2. Method

### 2.1. A Solder Layer Degradation Model Based on Baseplate Thermal Gradient Analysis

**Figure 1:**
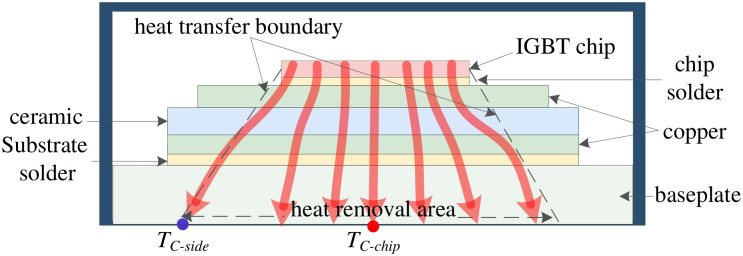
Schematic diagram of the heat flow inside a healthy power device (red arrows indicate heat flow).

The power device comprises multiple IGBT chips, which serve as the primary heat sources within the device. The heat generated on the surface of these IGBT chips propagates through several material layers before reaching the baseplate, as illustrated in Figure 1.

In consideration of the thermal diffusion characteristics associated with device heat dissipation, the temperature distribution area of the baseplate is more extensive compared to that of the chip. Given that a majority of the heat propagates downward along the optimal thermal path (i.e., in the vertical direction), the central region at the bottom surface of the baseplate exhibits significantly higher temperatures than other areas, leading to an uneven spatial temperature distribution across the baseplate. The extent of non-uniformity in the multi-point temperature distribution of the baseplate can be quantified using the temperature gradient ∇T as follows:

(1) $$\nabla T=\frac{T_{C-\mathrm{chip}}-T_{C-\mathrm{side}}}{d},$$

where $T_{C-\mathrm{chip}}$ and $T_{C-\mathrm{side}}$ denote the temperatures at spatial points selected on the baseplate, representing the central and edge regions of the baseplate, respectively. As illustrated in Figure 1, $T_{C-\mathrm{chip}}$ exhibits high sensitivity to alterations in the device’s thermal path due to solder layer aging, whereas $T_{C-\mathrm{side}}$ is situated in the initial region affected by solder layer aging. The variable *d* signifies the distance between these two selected points.

**Figure 2:**
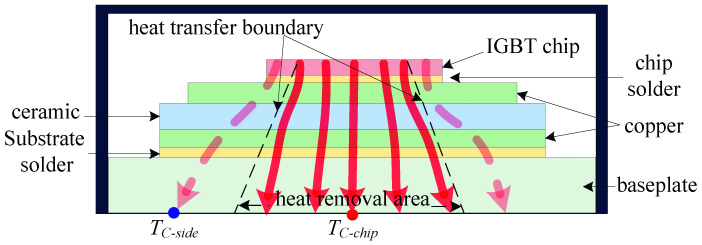
Schematic diagram of the heat flow inside a fatigued power device (red arrows indicate heat flow).

Fatigue cracks in the solder layer typically initiate at the corners due to stress concentration from the mismatch in coefficients of thermal expansion (CTE) between the chip, solder, and substrate. This phenomenon initiates crack formation at the edges of the solder layer, which progressively propagates towards the center. Consequently, the effective thermal diffusion area within the device is reduced, as illustrated in Figure 2. Heat generated by the chip can now only be dissipated through the intact central region of the solder layer. Under identical operating conditions, when fatigue aging of the solder layer occurs exclusively, the temperature in the central area of the substrate continuously increases, whereas the temperature in the surrounding regions steadily decreases. When the aging of the solder layer and the bond wires occurs concurrently, the degradation of the bond wires results in increased power losses. This counteracts the temperature reduction in the remaining areas of the baseplate due to the aging of the solder layer, leading to a rise in temperature across both the center and the remaining areas of the baseplate. Notably, the aging of the solder layer causes a more pronounced increase in the temperature of the center area compared to the remaining areas, thereby exacerbating the temperature non-uniformity across the baseplate. Consequently, this leads to a continuous increase in the parameter ∇T.

To this end, the parameter ∇T is employed for the online monitoring of the solder layer’s fatigue aging process. To mitigate the impact of power loss on ∇T, it is normalized with respect to power loss, resulting in $\nabla T_{P}$.

(2) $$\nabla T_{P}=\frac{T_{C-\mathrm{chip}}-T_{C-\mathrm{side}}}{P_{\mathrm{tot}}\cdot d},$$

where $P_{\mathrm{tot}}$ denotes the total power loss of the device, this normalization facilitates a more precise evaluation of the solder layer’s condition by accounting for variations in power loss that might otherwise influence monitoring outcomes. The value of $P_{\mathrm{tot}}$ can be determined using the following formula:

(3) $$P_{\mathrm{tot}}=\frac{T_{C-\mathrm{chip}}-T_{H}}{Z_{\mathrm{CH}}},$$

where $Z_{\mathrm{CH}}$ and $T_{H}$ denote the thermal resistance between the device baseplate and the heat sink, and the temperature at the heat sink, respectively, as illustrated in Figure 2.

The thermal resistance between the baseplate and heatsink ($Z_{\mathrm{CH}}$) is treated as a constant in this model. This is a valid assumption for the service life of IGBT modules in rail traction applications, where high-stability thermal interface materials (e.g., sintered silver pastes) are typically used, and mounting pressure is maintained by spring clips. Furthermore, any gradual increase in $Z_{\mathrm{CH}}$ due to TIM aging would manifest as a uniform temperature rise across the entire baseplate, which has a minimal effect on the spatially differential metric $\nabla T_{P}$. For applications where TIM degradation is a significant concern, the model can be augmented by monitoring the absolute temperature difference ($T_{C-\mathrm{chip}}-T_{H}$) alongside $\nabla T_{P}$ to decouple the effects.

The thermal impedance value $Z_{\mathrm{JC}}$ from the IGBT chip to the baseplate serves as a critical parameter for quantifying the degree of aging in the device’s solder layer. The methodology for its calculation is outlined below:

(4) $$Z_{\mathrm{JC}}=\frac{T_{J}-T_{C-\mathrm{chip}}}{P_{\mathrm{tot}}}.$$

The calculation of $Z_{\mathrm{JC}}$ is contingent upon the chip junction temperature $T_{J}$. However, obtaining an accurate measurement of $T_{J}$ in industrial settings presents significant challenges. Given that variations in both $\nabla T_{P}$ and $Z_{\mathrm{JC}}$ are primarily attributed to the fatigue aging of the solder layer, a database correlating $\nabla T_{P}$ and $Z_{\mathrm{JC}}$ has been established, using the degree of solder aging as an intermediate variable. In field applications, $Z_{\mathrm{JC}}$ can be inferred from $\nabla T_{P}$ to evaluate the aging condition of the device’s solder layer. For practical implementation, an offline accelerated aging test method can be employed to construct a comprehensive database encompassing $\nabla T_{P}$ and $Z_{\mathrm{JC}}$ data. The detailed methodology for establishing this database will be elaborated in the experimental section.

**Figure 3:**
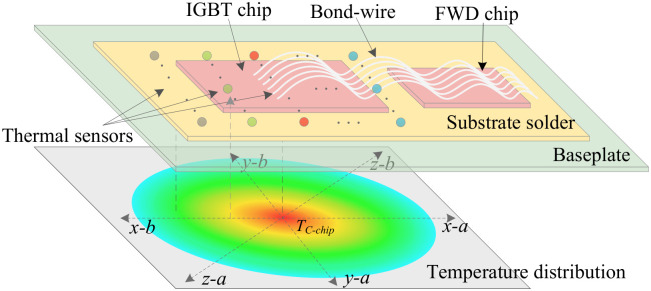
Schematic diagram of a two-dimensional temperature-based monitoring model (colored circles represent temperature measurement points, and the ellipsis indicates the continuation of the pattern).

For applications with stringent reliability requirements, such as high-speed train traction converters, a two-dimensional temperature-based monitoring model can be implemented to assess the aging of the solder layer. The evolution of baseplate temperature is monitored in six directions derived from the three primary axes (*x*, *y*, and *z*) on the two-dimensional plane of the baseplate, as illustrated in Figure 3. These directions are denoted as x−1, x−2, y−1, y−2, z−1, and z−2. The temperature gradients $\nabla T_{P,x-1}$, $\nabla T_{P,x-2}$, $\nabla T_{P,y-1}$, $\nabla T_{P,y-2}$, $\nabla T_{P,z-1}$, and $\nabla T_{P,z-2}$ represent the temperature differences on the lower surface of the baseplate in these six directions, as shown in the following equations:

(5) $$\left\{\begin{array}{c} \nabla T_{P,x-1}=\frac{T_{C-\mathrm{chip}}-T_{C-\mathrm{side},x-1}}{P_{\mathrm{tot}}\cdot d_{x-1}},\\ \nabla T_{P,x-2}=\frac{T_{C-\mathrm{chip}}-T_{C-\mathrm{side},x-2}}{P_{\mathrm{tot}}\cdot d_{x-2}},\\ \nabla T_{P,y-1}=\frac{T_{C-\mathrm{chip}}-T_{C-\mathrm{side},y-1}}{P_{\mathrm{tot}}\cdot d_{y-1}},\\ \nabla T_{P,y-2}=\frac{T_{C-\mathrm{chip}}-T_{C-\mathrm{side},y-2}}{P_{\mathrm{tot}}\cdot d_{y-2}},\\ \nabla T_{P,z-1}=\frac{T_{C-\mathrm{chip}}-T_{C-\mathrm{side},z-1}}{P_{\mathrm{tot}}\cdot d_{z-1}},\\ \nabla T_{P,z-2}=\frac{T_{C-\mathrm{chip}}-T_{C-\mathrm{side},z-2}}{P_{\mathrm{tot}}\cdot d_{z-2}}. \end{array}\right.$$

The temperature gradients of the baseplate in the principal directions are denoted as $\nabla T_{P,x}$, $\nabla T_{P,y}$, and $\nabla T_{P,z}$, as presented in the following equations:

(6) $$\left\{\begin{array}{c} \nabla T_{P,x}=\frac{\nabla T_{P,x-1}+\nabla T_{P,x-2}}{2},\\ \nabla T_{P,y}=\frac{\nabla T_{P,y-1}+\nabla T_{P,y-2}}{2},\\ \nabla T_{P,z}=\frac{\nabla T_{P,z-1}+\nabla T_{P,z-2}}{2}. \end{array}\right.$$

And the value of $\nabla T_{P}$ is estimated as follows:

(7) $$\begin{array}{c} \nabla T_{P}=\frac{\nabla T_{P,x}+\nabla T_{P,y}+\nabla T_{P,z}}{3}. \end{array}$$

By utilizing the parameter $\nabla T_{P}$, online monitoring of solder layer aging can be achieved irrespective of the location of defects such as cracks within the solder layer.

### 2.2. A Bond Wire Aging Model Based on Electrical Parameter Inversion

Bond wire lift-off represents an additional failure mode in the packaging of IGBT modules. The mismatch in coefficients of thermal expansion between the bond wire and the chip induces stress at their interface during temperature fluctuations. This stress, when continuously applied, results in crack formation at the connection point, thereby increasing the impedance between the collector and emitter. Consequently, this leads to an elevation in the device’s on-state voltage $V_{\mathrm{ce},\mathrm{on}}$. Therefore, monitoring $V_{\mathrm{ce},\mathrm{on}}$ is a common method for assessing the aging condition of bond wires. However, the variations in $V_{\mathrm{ce},\mathrm{on}}$ caused by bond wire aging are on the millivolt scale, which necessitates the development of a specialized high-precision measurement circuit. This inevitably introduces additional complexity into the power conversion control circuit, leading to increased costs for bond wire aging monitoring and potentially impacting the normal operation of the energy conversion system. Therefore, this paper proposes a $V_{\mathrm{ce},\mathrm{on}}$ measurement model based on the inverse thermal parameter algorithm.

$V_{\mathrm{ce},\mathrm{on}}$ is a critical parameter for assessing the conduction losses of power devices. An elevation in $V_{\mathrm{ce},\mathrm{on}}$ results in a corresponding increase in conduction losses. Consequently, $V_{\mathrm{ce},\mathrm{on}}$ can be inversely determined based on the measured conduction losses. The conduction losses of the power device are calculated as follows:

(8) $$P_{\mathrm{cond}}=V_{\mathrm{ce},\mathrm{on}}\cdot I_{C}\cdot D,$$

where $P_{\mathrm{cond}}$ represents the conduction loss, $I_{C}$ denotes the load current, and *D* is the duty cycle. The load current can be acquired from sensor measurements, while the duty cycle can be determined from the control strategy.

The total power consumption of the power device comprises both conduction losses and switching losses.

(9) $$P_{\mathrm{tot}}=P_{\mathrm{cond}}+P_{\mathrm{sw}},$$

where $P_{\mathrm{tot}}$ denotes the total power consumption of the power device, as given by (3). $P_{\mathrm{sw}}$ represents the switching loss of the power device, which can be derived from the subsequent equation.

(10) $$P_{\mathrm{sw}}=\left(E_{\mathrm{on}}+E_{\mathrm{off}}\right)\cdot f,$$

where $E_{\mathrm{on}}$ represents the turn-on energy and $E_{\mathrm{off}}$ denotes the turn-off energy of the device, while *f* signifies the switching frequency. By applying Equations (3) and (10), the total power consumption and switching losses of the power device can be determined. Subsequently, substituting these values into Equation (9) enables the calculation of the conduction losses of the power device.

The turn-on and turn-off energies of power devices are influenced by factors such as DC voltage, gate resistance, and chip junction temperature. The expressions for $E_{\mathrm{on}}$ and $E_{\mathrm{off}}$ can be derived as follows.

(11) $$\begin{array}{c} E_{\mathrm{on}}=\left(\alpha_{\mathrm{on}}I_{C}+\beta_{\mathrm{on}}\right)\frac{R_{G}}{R_{G,\mathrm{test}}}\cdot \frac{V_{\mathrm{DC}}}{V_{\mathrm{DC},\mathrm{test}}}\cdot \frac{E_{\mathrm{on}}\left(T_{J}\right)}{E_{\mathrm{on}}\left(T_{J,\max}\right)},\\ E_{\mathrm{off}}=\left(\alpha_{\mathrm{off}}I_{C}+\beta_{\mathrm{off}}\right)\frac{R_{G}}{R_{G,\mathrm{test}}}\cdot \frac{V_{\mathrm{DC}}}{V_{\mathrm{DC},\mathrm{test}}}\cdot \frac{E_{\mathrm{off}}\left(T_{J}\right)}{E_{\mathrm{off}}\left(T_{J,\max}\right)}, \end{array}$$

where $\alpha_{\mathrm{on}}$, $\alpha_{\mathrm{off}}$, $\beta_{\mathrm{on}}$, and $\beta_{\mathrm{off}}$ are the coefficients for the turn-on and turn-off energies of the power device, $E_{\mathrm{on}}\left(T_{J,\max}\right)$ and $E_{\mathrm{off}}\left(T_{J,\max}\right)$ denote the turn-on and turn-off energies at the chip’s rated maximum junction temperature, respectively. These values can be obtained from the manufacturer’s datasheet. $E_{\mathrm{on}}\left(T_{J}\right)$ and $E_{\mathrm{off}}\left(T_{J}\right)$ represent the turn-on and turn-off energies at a specific junction temperature of the power device. $R_{G}$ is the gate resistance value of the power device, while $R_{G,\mathrm{test}}$ is the gate resistance used by the manufacturer during testing. $V_{\mathrm{DC}}$ represents the load voltage across the power device during operation, whereas $V_{\mathrm{DC},\mathrm{test}}$ is the voltage utilized by the manufacturer during testing. The impact of varying operating conditions on $E_{\mathrm{on}}$ and $E_{\mathrm{off}}$ can be mitigated through the proportional relationship between voltage, resistance, and energy.

While bond wire lift-off can slightly alter parasitic package inductance, its impact on switching energy is secondary compared to the dominant influence of junction temperature and load current. The experimental results in Section 4.4 demonstrate that using the pre-characterized switching loss model of a healthy device within the inversion algorithm yields accurate $V_{\mathrm{ce},\mathrm{on}}$ estimates throughout the bond wire aging process, validating the practical robustness of this approach for condition monitoring.

**Figure 4:**
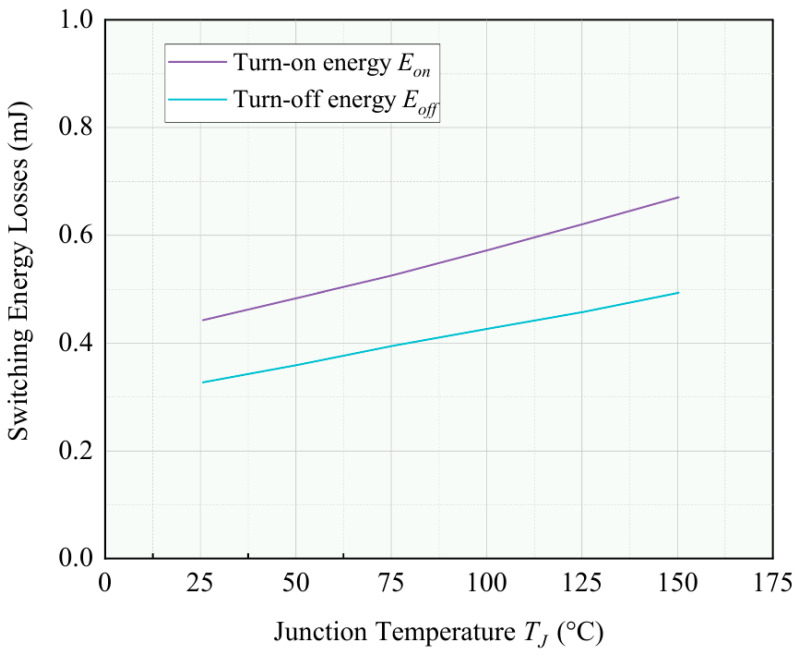
Illustrative example of the relationship between the switching energies ($E_{\mathrm{on}}$, $E_{\mathrm{off}}$) and the junction temperature ($T_{J}$) based on a commercial IGBT datasheet.

**Figure 5:**
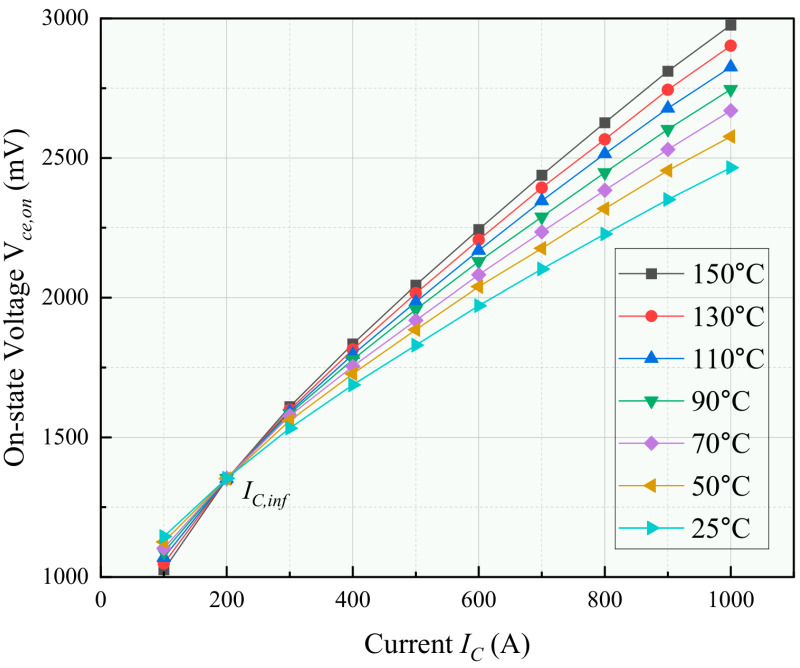
Illustrative example of the I–V characteristics of a power device at varying $T_{J}$ based on a commercial IGBT datasheet.

The relationships shown in Figure 4 and Figure 5 are illustrative examples based on a commercial IGBT datasheet. In our implementation, the specific coefficients for the switching loss model ($\alpha_{\mathrm{on}}$, $\beta_{\mathrm{on}}$, etc.) and the inflection point current ($I_{C,\inf}$) were determined through characterization tests on the actual device under test (SEMIKRON SKM75GB12T4, SEMIKRON, Nuremberg, Germany), as detailed in Section 4.2.

The relationship between the turn-on energy $E_{\mathrm{on}}$, turn-off energy $E_{\mathrm{off}}$, and chip junction temperature $T_{J}$ can be determined through offline testing. For instance, using Infineon’s SGP20N60 power device (Infineon Technologies AG, Munich, Germany), Figure 4 illustrates the relationship between $E_{\mathrm{on}}$, $E_{\mathrm{off}}$, and the chip junction temperature $T_{J}$. As observed in Figure 4, there is a nearly linear relationship between $E_{\mathrm{on}}$, $E_{\mathrm{off}}$, and $T_{J}$. By applying the method of least squares to fit the data, we derive the functional relationships between $E_{\mathrm{on}}$, $E_{\mathrm{off}}$, and $T_{J}$. In practical applications, substituting the measured chip junction temperature into these functions allows for the calculation of $E_{\mathrm{on}}\left(T_{J}\right)$ and $E_{\mathrm{off}}\left(T_{J}\right)$.

**Figure 6:**
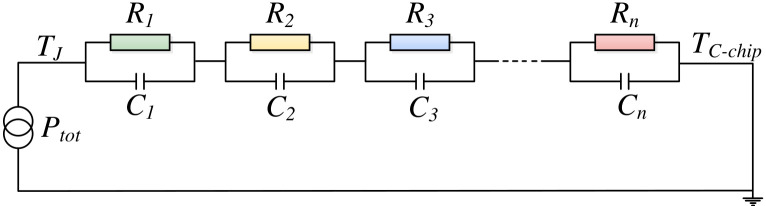
Schematic diagram of the Foster-type thermal network model.

From the analysis presented, it is evident that the chip junction temperature $T_{J}$ plays a crucial role in determining both $E_{\mathrm{on}}\left(T_{J}\right)$ and $E_{\mathrm{off}}\left(T_{J}\right)$. This study utilizes a Foster-type thermal network model to accurately measure $T_{J}$. As a thermal equivalent circuit model, the architecture of the Foster-type thermal network is illustrated in Figure 6.

The inputs to the Foster-type thermal network model include the total power losses of the power device, denoted as $P_{\mathrm{tot}}$, and the baseplate temperature, represented by $T_{C-\mathrm{chip}}$. The output of this model is the chip junction temperature, $T_{J}$. The internal structure of the Foster model comprises a series of thermal resistances and thermal capacitances. Accurate prediction of $T_{J}$ hinges on the precise identification of these thermal resistance and thermal capacitance parameters. The transient thermal impedance $Z_{\mathrm{JC}}\left(t\right)$, which characterizes the heat transfer from the chip to the baseplate, is a critical parameter that describes the internal thermal behavior of the power device. Its relationship with the thermal resistance and thermal capacitance parameters can be expressed as follows:

(12) $$Z_{\mathrm{JC}}\left(t\right)=\sum_{i=1}^{n}R_{i}\left(1-e^{-t/(R_{i}C_{i})}\right).$$

The transient thermal impedance curve can be obtained via finite element analysis or experimental testing. By applying the least squares method to fit the transient thermal impedance values into (12), the thermal resistance and capacitance parameters are determined, thereby completing the construction of the Foster thermal network model. However, it is important to note that these parameters are sensitive to fatigue and aging effects in the solder layer. When such degradation occurs, it is imperative to promptly update the parameters. The methods for updating these parameters are detailed in [18], and this paper will not delve into the specifics.

The value of $V_{\mathrm{ce},\mathrm{on}}$ can be determined through the thermal parameter inversion algorithm, thereby enabling the monitoring of bond-wire aging. However, it is crucial to recognize that $V_{\mathrm{ce},\mathrm{on}}$ is influenced not only by bond wire aging but also by parameters such as load current $I_{C}$ and chip junction temperature $T_{J}$. Therefore, to accurately isolate the variations in $V_{\mathrm{ce},\mathrm{on}}$ due to bond wire aging, the effects of $I_{C}$ and $T_{J}$ must be minimized. A common method involves comparing $V_{\mathrm{ce},\mathrm{on}}$ values under consistent operating conditions, specifically with stable $I_{C}$ and $T_{J}$. This requires the prior establishment of an $I_{C}$–$T_{J}$–$V_{\mathrm{ce},\mathrm{on}}$ database for a healthy power device. By retrieving the $V_{\mathrm{ce},\mathrm{on}}$ value corresponding to the current operating conditions from this database and comparing it with the measured $V_{\mathrm{ce},\mathrm{on}}$ value, the aging status of the bond-wire can be evaluated. Constructing such an $I_{C}$–$T_{J}$–$V_{\mathrm{ce},\mathrm{on}}$ database is resource-intensive, leading to increased costs associated with bond-wire monitoring. Therefore, developing an alternative solution that eliminates the need for this database remains a significant challenge.

The voltage $V_{\mathrm{ce},\mathrm{on}}$ is a critical characteristic parameter that indicates bond wire aging and is widely acknowledged as a temperature sensitive electrical parameter for monitoring the junction temperature. Using Infineon’s SGP20N60 power device (Munich, Germany) as a case study, Figure 5 illustrates the I–V characteristics of the device at various $T_{J}$ values. Analysis of the data in Figure 5 reveals a clear correlation between $V_{\mathrm{ce},\mathrm{on}}$ and $T_{J}$ under different load currents. Specifically, when the load current ($I_{C}$) exceeds 200 A, $V_{\mathrm{ce},\mathrm{on}}$ exhibits a positive correlation with $T_{J}$. Conversely, when $I_{C}$ is below 200 A, $V_{\mathrm{ce},\mathrm{on}}$ shows a negative correlation with $T_{J}$. At a load current of $I_{C}=200$ A, $V_{\mathrm{ce},\mathrm{on}}$ remains independent of $T_{J}$.

The point at which $I_{C}=200$ A is referred to as the inflection point, denoted as $I_{C,\inf}$, where the value of $V_{\mathrm{ce},\mathrm{on}}$ becomes independent of $T_{J}$. Studies by Singh et al. [13] have shown that this inflection point remains constant despite bond wire aging. Therefore, it is feasible to employ a thermal parameter inversion algorithm to determine the $V_{\mathrm{ce},\mathrm{on}}$ value at the inflection point and compare it with the $V_{\mathrm{ce},\mathrm{on}}$ value of a healthy power device, thereby assessing the degree of bond wire aging. This method allows for the monitoring of bond wire aging without the need to establish an extensive $I_{C}$–$T_{J}$–$V_{\mathrm{ce},\mathrm{on}}$ database, thus significantly reducing associated costs.

## 3. Implementation of the Proposed Method

**Figure 7:**
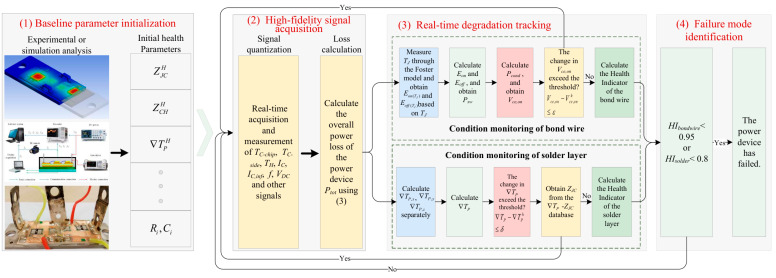
Schematic flowchart of the proposed method.

The proposed method is implemented through a structured online monitoring system, whose overall workflow is illustrated in Figure 7. The system operation can be conceptualized in four sequential phases: (1) Baseline Parameter Initialization: establishing reference metrics for a healthy device; (2) Multi-Modal Signal Acquisition: collecting real-time thermal and electrical data; (3) Degradation State Tracking: executing algorithms to monitor solder layer and bond wire health; (4) Failure Mode Identification & Prognostics: assessing health indices and triggering maintenance actions. The core of Phase (3) lies in the parallel execution of the two models introduced in Section 2. Their detailed implementation procedures are described below.

### 3.1. Implementation of the Solder Layer Degradation Monitoring Model

This subsection details the operational steps to realize the solder layer degradation model based on baseplate thermal gradient analysis (Section 2.1).

Step 1: Data Acquisition. Multiple temperature sensors (e.g., thermocouples) are deployed on the baseplate lower surface to capture its spatial temperature distribution. Key measurements include:

- The temperature at the central region beneath the chip, $T_{C-\mathrm{chip}}$.
- Temperatures at multiple peripheral points, $\left\{T_{C-\mathrm{side},k-i}\right\}$, where k∈{x,y,z} denotes the principal axis and *i* denotes the specific sensor along that axis (e.g., i=1,2 for two opposite directions), as conceptualized in Figure 3.
- The heat sink temperature, $T_{H}$.

Step 2: Power Loss Calculation. The total device power loss $P_{\mathrm{tot}}$ is calculated in real-time using (3): $P_{\mathrm{tot}}=\left(T_{C-\mathrm{chip}}-T_{H}\right)/Z_{\mathrm{CH}}$, where $Z_{\mathrm{CH}}$ is the constant thermal resistance between the baseplate and heat sink.

Step 3: Multi-Directional Gradient Computation. Following Equation (5), the normalized temperature gradient for each monitored direction is computed:

$$\nabla T_{P,k-i}=\frac{T_{C-\mathrm{chip}}-T_{C-\mathrm{side},k-i}}{P_{\mathrm{tot}}\cdot d_{k-i}},\;\left(k\in \left\{x,y,z\right\},\;i=1,2\right).$$

where $d_{k-i}$ is the distance between the central point and the specific peripheral point.

Step 4: Composite Gradient Index Derivation. The gradients along each principal axis are averaged using (6):

$$\nabla T_{P,k}=\frac{\nabla T_{P,k-1}+\nabla T_{P,k-2}}{2}.$$

The overall solder layer degradation index $\nabla T_{P}$ is then obtained by averaging the three principal axis gradients, as per (7):

$$\nabla T_{P}=\frac{\nabla T_{P,x}+\nabla T_{P,y}+\nabla T_{P,z}}{3}.$$

Step 5: Health Assessment. The calculated $\nabla T_{P}$ is compared against the healthy baseline $\nabla T_{P}^{h}$ established during initialization. A significant deviation indicates solder layer aging. The decision logic is:

(13) $$\mathrm{If}\;|\nabla T_{P}-\nabla T_{P}^{h}|>\delta ,\;\mathrm{then}\;\mathrm{compute}\;\mathrm{Health}\;\mathrm{Index}\;\left(\mathrm{HI}\right):\;{\mathrm{HI}}_{\mathrm{solder}}=1-\frac{Z_{\mathrm{JC}}-Z_{\mathrm{JC}}^{h}}{Z_{\mathrm{JC}}^{h}}.$$

The failure threshold ${\mathrm{HI}}_{\mathrm{solder}}\leq 0.8$ (corresponding to a 20% increase in $Z_{\mathrm{JC}}$) is aligned with the common industry end-of-life criterion for solder joint reliability. The value of $Z_{\mathrm{JC}}$ corresponding to the current $\nabla T_{P}$ is retrieved from the pre-established $\nabla T_{P}$–$Z_{\mathrm{JC}}$ database (constructed via offline aging tests). If ${\mathrm{HI}}_{\mathrm{solder}}\leq 0.8$, imminent solder failure is signaled, prompting component replacement. Otherwise (${\mathrm{HI}}_{\mathrm{solder}}>0.8$), the system continues monitoring and may trigger model parameter recalibration.

### 3.2. Implementation of the Bond Wire Aging Monitoring Model

This subsection details the steps to realize the bond wire aging model based on electrical parameter inversion (Section 2.2). The key innovation is inferring $V_{\mathrm{ce},\mathrm{on}}$ at the current inflection point ($I_{C,\inf}$) without needing a full $I_{C}$–$T_{J}$–$V_{\mathrm{ce},\mathrm{on}}$ database.

Step 1: Junction Temperature Estimation. A Foster-type thermal network model (Figure 6) is used. It takes the measured $T_{C-\mathrm{chip}}$ and the calculated $P_{\mathrm{tot}}$ as inputs to estimate the chip junction temperature $T_{J}$:

$$T_{J}=F\left(T_{C-\mathrm{chip}},P_{\mathrm{tot}}\right),$$

where F(·) represents the Foster model transfer function. The model parameters ($R_{i}$, $C_{i}$) are identified offline via curve fitting to the transient thermal impedance $Z_{\mathrm{JC}}\left(t\right)$ (12) and are updated online if significant solder layer aging is detected.

Step 2: Switching Loss Calculation. The switching energies $E_{\mathrm{on}}$ and $E_{\mathrm{off}}$ at the estimated $T_{J}$ are computed using (11):

$$E_{\mathrm{on}/\mathrm{off}}\left(T_{J}\right)=\left(\alpha_{\mathrm{on}/\mathrm{off}}I_{C}+\beta_{\mathrm{on}/\mathrm{off}}\right)\cdot \frac{R_{G}}{R_{G,\mathrm{test}}}\cdot \frac{V_{\mathrm{DC}}}{V_{\mathrm{DC},\mathrm{test}}}\cdot \frac{\Psi_{\mathrm{on}/\mathrm{off}}\left(T_{J}\right)}{\Psi_{\mathrm{on}/\mathrm{off}}\left(T_{J,\max}\right)},$$

where $\Psi_{\mathrm{on}/\mathrm{off}}\left(T_{J}\right)$ represents the temperature-dependent scaling factor derived from offline characterization data (as in Figure 4). The total switching loss $P_{\mathrm{sw}}$ is then obtained via (10): $P_{\mathrm{sw}}=\left(E_{\mathrm{on}}+E_{\mathrm{off}}\right)\cdot f$.

Step 3: Conduction Loss and $V_{\mathrm{ce},\mathrm{on}}$ Inversion. The conduction loss $P_{\mathrm{cond}}$ is derived from the power balance in (9):

$$P_{\mathrm{cond}}=P_{\mathrm{tot}}-P_{\mathrm{sw}}.$$

Crucially, $V_{\mathrm{ce},\mathrm{on}}$ is calculated at the inflection point load current $I_{C,\inf}$ (where $V_{\mathrm{ce},\mathrm{on}}$ is independent of $T_{J}$). Assuming the device operates at or can be momentarily controlled to $I_{C,\inf}$ with duty cycle *D*, the on-state voltage is inverted using (8):

$$V_{\mathrm{ce},\mathrm{on}}=\frac{P_{\mathrm{cond}}}{I_{C,\inf}\cdot D}.$$

Step 4: Health Assessment. The inferred $V_{\mathrm{ce},\mathrm{on}}$ at $I_{C,\inf}$ is compared to the healthy baseline value $V_{\mathrm{ce},\mathrm{on}}^{h}$:

(14) $$\mathrm{If}\;|V_{\mathrm{ce},\mathrm{on}}-V_{\mathrm{ce},\mathrm{on}}^{h}|\;>\;\varepsilon ,\;\mathrm{then}\;\mathrm{compute}\;\mathrm{Health}\;\mathrm{Index}:\;\;{\mathrm{HI}}_{\mathrm{bond}\;\mathrm{wire}}=1-\frac{V_{\mathrm{ce},\mathrm{on}}-V_{\mathrm{ce},\mathrm{on}}^{h}}{V_{\mathrm{ce},\mathrm{on}}^{h}}.$$

If ${\mathrm{HI}}_{\mathrm{bond}\;\mathrm{wire}}\leq 0.95$, significant bond wire degradation is indicated, activating failure mitigation protocols. Otherwise (${\mathrm{HI}}_{\mathrm{bond}\;\mathrm{wire}}>0.95$), continuous monitoring is maintained.

### 3.3. System Integration and Output

The monitoring system integrates the outputs of the two parallel models. The degradation state vector $\Theta ={\left[Z_{\mathrm{JC}},V_{\mathrm{ce},\mathrm{on}},\nabla T_{P}\right]}^{T}$ is continuously updated. The health indices ${\mathrm{HI}}_{\mathrm{solder}}$ and ${\mathrm{HI}}_{\mathrm{bond}\;\mathrm{wire}}$ provide quantitative measures for prognostic health management, enabling predictive maintenance and preventing catastrophic failures.

## 4. Experimental Verification

To comprehensively validate the proposed dual-model health monitoring framework, an accelerated power cycling test platform was constructed. The platform was designed to simultaneously induce and monitor solder layer and bond wire degradation under controlled, repetitive thermal stress. Unlike a simple DC conduction test, this setup incorporates switching operation to generate realistic power loss profiles (both conduction and switching losses), which is crucial for validating the thermal–electrical inverse calculation model for bond wire monitoring.

### 4.1. Experimental Setup and Device Under Test

The core Device Under Test (DUT) was a SEMIKRON IGBT module. To enable precise thermal monitoring of the baseplate and chip, the upper silicone gel encapsulation was carefully removed, leaving the internal die, bond wires, and substrate intact. The DUT was mounted on a custom aluminum heatsink with machined grooves for embedding K-type thermocouples to measure baseplate temperatures ($T_{C-\mathrm{chip}}$, $T_{C-\mathrm{side}}$) and heatsink temperature ($T_{H}$) with minimal interference.

For the purpose of this controlled laboratory experiment, K-type thermocouples were directly attached to the baseplate. In a field-deployed module, electrical insulation of contact sensors is mandatory. This can be achieved by using sensors with integrated insulation or, more practically, by employing non-contact infrared temperature sensors aimed at the baseplate surface. The proposed monitoring algorithm is compatible with both contact (with proper insulation) and non-contact temperature measurement techniques.

The test platform featured synchronized, high-fidelity measurement systems:

- **Thermal Measurement**: K-type thermocouples connected to a HIOKI MR8875-30 DAQ system (HIOKI E.E. Corporation, Ueda, Japan) provided baseplate and heatsink temperatures. A Fortic 615C infrared camera (±1 °C accuracy) was used to directly measure the chip junction temperature ($T_{J}$) for independent validation of the Foster model output.
- **Electrical Measurement**: The HIOKI MR8875-30 DAQ system, equipped with high-precision voltage probes captured the collector-emitter voltage ($V_{\mathrm{ce}}$) and a current sensor measured the load current ($I_{C}$). This provided the ground truth $V_{\mathrm{ce},\mathrm{on}}$ for comparison against the model-predicted value.
- **Control & Power**: A Tektronix AFG1022 signal generator (Tektronix, Inc., Beaverton, OR, USA) and a gate driver controlled the switching of the DUT. A DC power supply and an electronic load were used to apply the desired electrical stress.

All instruments were synchronized via the DAQ system to ensure temporal alignment of thermal and electrical data, which is essential for the inverse calculation.

### 4.2. Pre-Test Characterization and Baseline Establishment

**Figure 8:**
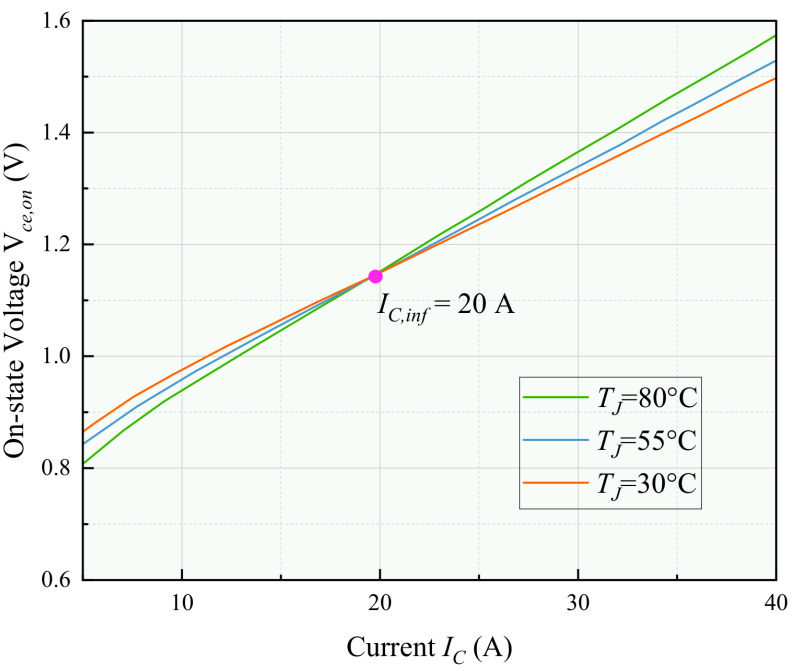
Experimental results of the I–V characteristics of the given power device at varying $T_{J}$.

Before aging, key device characteristics were established under healthy conditions:

1. **I–V Characterization and $I_{C,\inf}$ Determination**: Using a pulsed current method at controlled case temperatures, the $I_{C}$–$V_{\mathrm{ce},\mathrm{on}}$ curves at multiple junction temperatures were measured. As shown in Figure 8, the intersection point (inflection point) where $V_{\mathrm{ce},\mathrm{on}}$ is invariant with $T_{J}$ was identified as $I_{C,\inf}$ ≈ 20 A for the DUT.
2. **Baseline Parameter Database**:
  - **Solder Model**: The healthy baseline thermal gradient $\nabla T_{P}^{h}$ and chip-to-case thermal impedance $Z_{\mathrm{JC}}^{h}$ were recorded.
  - **Bond Wire Model**: The healthy $V_{\mathrm{ce},\mathrm{on}}^{h}$ at $I_{C,\inf}$ was measured and recorded.
  - **Foster Model Parameters**: The transient thermal impedance curve $Z_{\mathrm{JC}}\left(t\right)$ was characterized, and the Foster model parameters ($R_{i}$, $C_{i}$) were extracted via curve fitting of (12).

### 4.3. Accelerated Power Cycling Test Procedure

**Figure 9:**
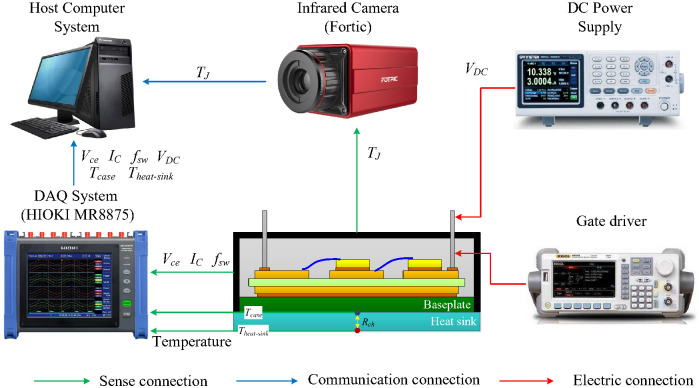
Schematic of the experimental setup.

The DUT was subjected to an accelerated power cycling test designed to induce both solder fatigue and bond wire heel cracking. The test profile was executed by the circuit illustrated in Figure 9.

The test procedure was designed to replicate realistic operating conditions while accelerating aging:

- **Switching Conditions:** To simulate actual IGBT operation in traction converters, the device was switched at 1kHz with a 50% duty cycle during conduction periods, generating both conduction and switching losses.
- **Thermal Cycling Protocol:** Power cycling was achieved by periodically enabling and disabling the test circuit using an external control switch. Each cycle consisted of a 30-s ON period (during which the IGBT switched at 1 kHz with $I_{C}=50\;A$) followed by a 60-s OFF period. This 90-s cycle induced a junction temperature swing $\Delta T_{J}$ of approximately 50 °C (from 50 °C to 100 °C), as measured by the infrared camera.
- **Waveform Characteristics:** The load current waveform exhibits a macroscopic square wave with 30-s ON and 60-s OFF periods. During the ON periods, the current is further modulated by the 1 kHz switching of the IGBT.

The monitoring algorithm processes instantaneous measurements and is therefore capable of handling the varying temperatures encountered in real mission profiles. The health indicators ($\nabla T_{P}$ and $V_{\mathrm{ce},\mathrm{on}}$) are updated in real-time, providing continuous condition assessment.

The test continued until a failure criterion—a 20% increase in $Z_{\mathrm{JC}}$ or a 5% increase in $V_{\mathrm{ce},\mathrm{on}}$ at $I_{C,\inf}$—was met.

### 4.4. Experimental Results and Analysis

**Figure 10:**
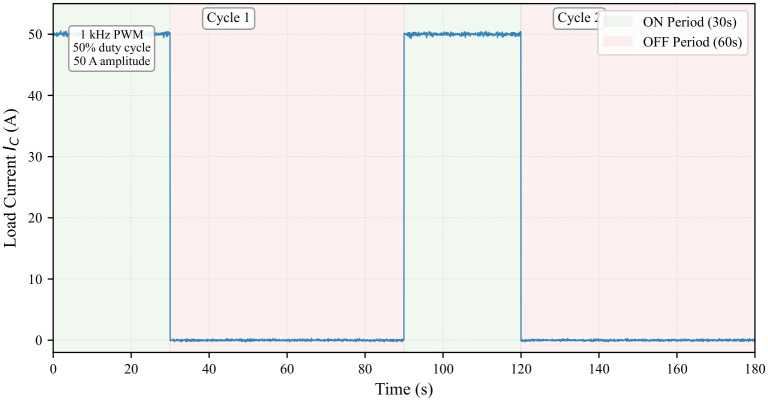
Schematic representation of the power cycling current waveform. The 30-s ON periods (green shaded) consist of 1 kHz PWM switching between 0 A and 50 A (50% duty cycle). The high switching frequency results in a filled-band appearance during ON periods. The 60-s OFF periods (red shaded) show zero current (blue line indicates the current curve applied to the IGBT module.).

**Figure 11:**
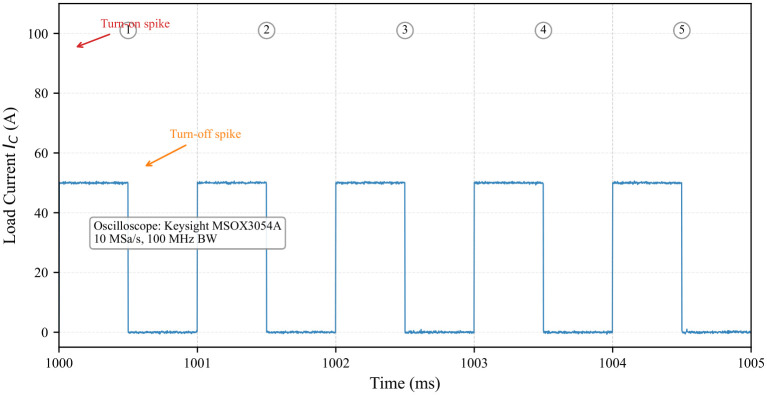
Measured PWM current waveform detail during an ON period, showing five switching cycles (labeled 1–5) with realistic turn-on and turn-off spikes. The measurement was performed at 10 MSa/s using a oscilloscope.

**Figure 12:**
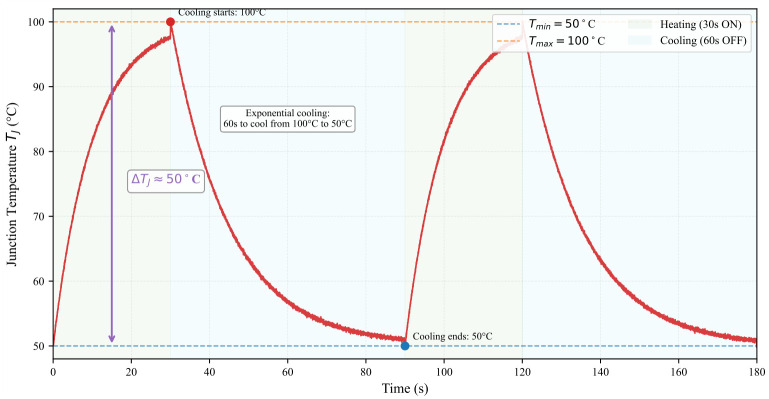
Measured junction temperature cycling during power cycling. The temperature rises from 50 °C to 100 °C during the 30-s ON periods and falls from 100 °C to 50 °C during the 60-s OFF periods.

The experimental waveforms are illustrated in Figure 10, Figure 11 and Figure 12. Due to the high switching frequency (1 kHz) during the ON periods, the individual PWM cycles cannot be resolved at the macroscopic time scale of the complete cycling test (180 s total duration). The 30-s ON periods contain 30,000 individual PWM cycles, creating a filled-band appearance when viewed at the macroscopic scale. Therefore, Figure 10 serves as a schematic representation to illustrate the macroscopic cycling behavior. Figure 11 provides a detailed view at the appropriate time scale (5 ms window), clearly showing the actual measured PWM switching with realistic transients. Figure 12 shows the corresponding measured temperature cycling with exponential characteristics.

#### 4.4.1. Solder Layer Degradation Monitoring

The evolution of the solder layer was tracked using the method in Section 3.1. For each characterization phase, the spatial temperature gradients $\nabla T_{P}$ were calculated. Concurrently, the actual $Z_{\mathrm{JC}}$ was determined using the IR camera-measured $T_{J}$ and the calculated $P_{\mathrm{tot}}$ in (4).

**Figure 13:**
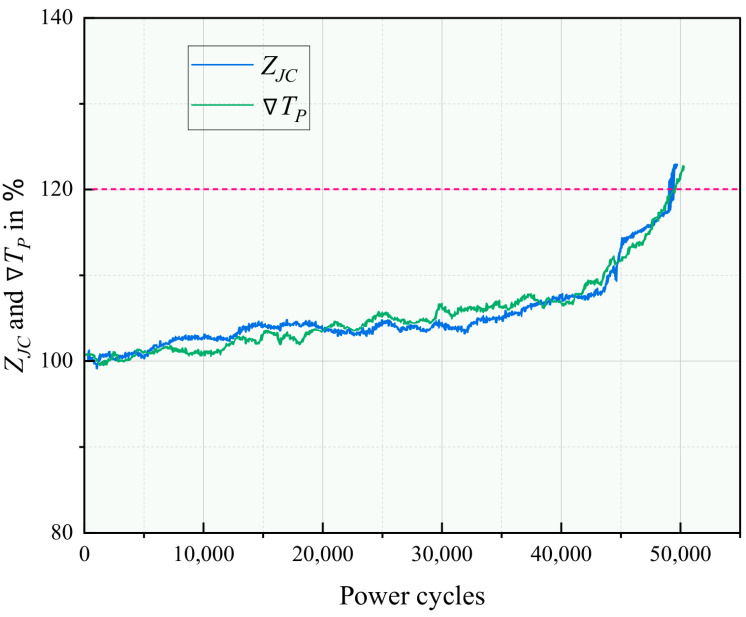
Experimental results of the evolution of the chip-to-case thermal impedance $Z_{\mathrm{JC}}$ and the normalized baseplate temperature gradient $\nabla T_{P}$ during the power cycling test on DUT-1. The red dashed line indicates the failure threshold (20% increase in $Z_{\mathrm{JC}}$ and $\nabla T_{P}$), at which the IGBT module is considered failed.

Figure 13 shows the correlated progression of $\nabla T_{P}$ and $Z_{\mathrm{JC}}$ over the number of cycles. Two distinct phases are observed:

- **Phase I (0–40k cycles):** Both $\nabla T_{P}$ and $Z_{\mathrm{JC}}$ increase gradually. This corresponds to the initiation and slow propagation of solder cracks from the edges.
- **Phase II (After 40k cycles):** A sharp acceleration in the growth of both parameters occurs. This is attributed to two compounding factors: (1) solder cracks propagating into the central heat path, drastically increasing $Z_{\mathrm{JC}}$; (2) a concurrent rapid increase in $V_{\mathrm{ce},\mathrm{on}}$ leading to higher power losses, which further exacerbates the temperature gradient $\nabla T_{P}$.

The strong correlation between $\nabla T_{P}$ and $Z_{\mathrm{JC}}$ validates the core premise of the solder monitoring model. The pre-established $\nabla T_{P}$–$Z_{\mathrm{JC}}$ database enables the online estimation of $Z_{\mathrm{JC}}$ from the easily measured $\nabla T_{P}$ in field applications.

#### 4.4.2. Bond Wire Degradation Monitoring

The effectiveness of the thermal–electrical inverse model was validated by comparing its predicted $V_{\mathrm{ce},\mathrm{on}}$ at $I_{C,\inf}$ against the DAQ-measured ground truth. The online implementation followed the steps outlined in Section 3.2 for each characterization dataset.

**Figure 14:**
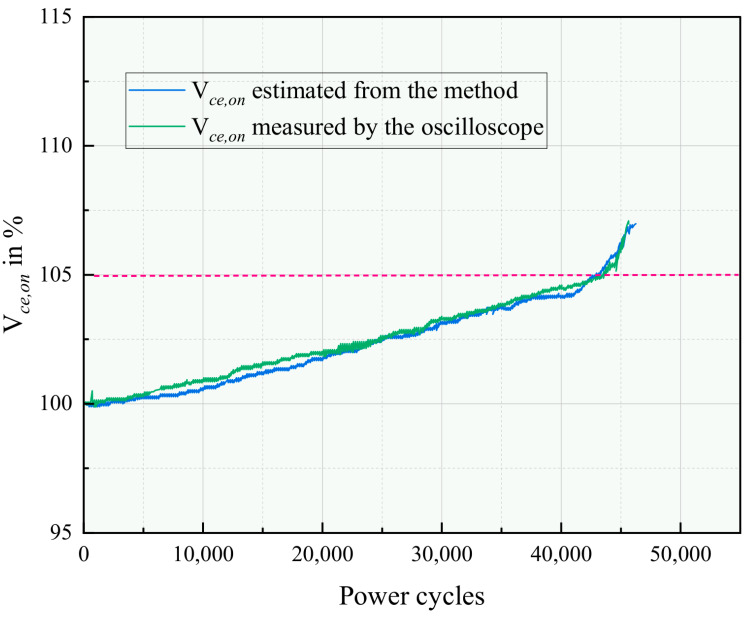
Experimental results of the comparison between the predicted and directly measured $V_{\mathrm{ce},\mathrm{on}}$ during the power cycling test. The red dashed line indicates the failure threshold (5% increase in $V_{\mathrm{ce},\mathrm{on}}$), at which the IGBT module is considered failed.

Figure 14 presents the results. The model-predicted $V_{\mathrm{ce},\mathrm{on}}$ shows excellent agreement with the directly measured values throughout the aging process. The trend reveals:

- **Linear Degradation Phase (0–40k cycles):** $V_{\mathrm{ce},\mathrm{on}}$ increases linearly, indicating progressive bond wire heel cracking.
- **Accelerated Failure Phase (After 40k cycles):** The rate of $V_{\mathrm{ce},\mathrm{on}}$ increase rises sharply, corresponding to the final stage of bond wire lift-off.

The discrepancy between the predicted and measured $V_{\mathrm{ce},\mathrm{on}}$ values remains within ±3.5 mV (±2.1%) throughout the test. The primary sources of uncertainty are the temperature measurement accuracy of the thermocouples (±1.5 °C), the fitting error of the Foster model (estimated ±2 °C in $T_{J}$), and the tolerances of the switching loss model coefficients. A Monte Carlo error propagation analysis confirms that the combined standard uncertainty for the inverted $V_{\mathrm{ce},\mathrm{on}}$ is below 3%, which is sufficient for detecting the millivolt-level shifts indicative of bond wire degradation.

### 4.5. Repeatability and Statistical Analysis

To assess the repeatability and statistical significance of the proposed method, the accelerated power cycling test was originally conducted on three IGBT modules (DUT-1, DUT-2, and DUT-3) of the same type (SEMIKRON SKM75GB12T4). All test conditions (load current, switching frequency, thermal cycle) were identical. This section presents the consolidated results from all three devices. The evolution of the key degradation indicators, $\nabla T_{P}$ and $V_{\mathrm{ce},\mathrm{on}}$ (at $I_{C,\inf}$), was tracked across all DUTs and exhibited consistent trends. The number of cycles to reach a 20% increase in $Z_{\mathrm{JC}}$ had a standard deviation of 8.5% across the three devices, and the rate of $V_{\mathrm{ce},\mathrm{on}}$ increase showed a standard deviation of 6.2%. This confirms the repeatability of the degradation process and the consistency of the monitoring signals.

### 4.6. Discussion and Practical Implications

The experimental results confirm that the proposed integrated framework can accurately monitor both critical failure modes in IGBT modules.

- The solder layer model provides a non-invasive way to track thermal impedance degradation via baseplate temperature gradients.
- The bond wire model demonstrates a cost-effective and reliable method to infer the key aging parameter $V_{\mathrm{ce},\mathrm{on}}$ through thermal parameter inversion, avoiding the need for invasive voltage sensing.
- The accelerated test under switching conditions proves the models’ validity in a realistic operational scenario involving both conduction and switching losses.

The synergy between the models is also evident: the bond wire degradation (increased $V_{\mathrm{ce},\mathrm{on}}$) leads to higher power losses, which in turn accelerates solder layer degradation, as captured in the later stages of the test. This validates the need for the concurrent monitoring approach proposed in this work. The method, relying primarily on temperature and standard electrical measurements, offers a practical, low-cost, and highly reliable solution for online health monitoring of power modules in critical applications.

## 5. Conclusions

To mitigate thermal degradation in IGBT modules induced by cyclic thermo-mechanical stresses, this study presents a novel methodology for real-time condition monitoring of power semiconductor devices. The method, relying primarily on temperature and standard electrical measurements, offers a practical, low-cost, and highly reliable solution for online health monitoring of power modules in critical applications, moving beyond the limitations of laboratory-only testing. The experimental configuration involves optimized placement of temperature transducers at the baseplate-heatsink interface to acquire high-resolution spatial temperature distributions characteristics. Through thermal gradient analysis, the temperature gradient ($▿T_{P}$) is quantitatively characterized as a diagnostic indicator for solder layer degradation monitoring. A systematic experimental protocol was implemented to establish a thermal impedance correlation database through accelerated power cycling experiments. The junction to case thermal impedance ($Z_{JC}$) was subsequently derived through multivariate regression analysis with $▿T_{P}$ as the independent variable. This empirical relationship enables inverse determination of $Z_{JC}$ for precise quantification of solder layer degradation states. The power loss decomposition methodology incorporates three-dimensional thermal network modeling. Total module losses were computed through heatsink thermal impedance characterization, with switching losses determined via switching characteristics of IGBT. Conduction losses were subsequently isolated through power analysis, enabling inverse computation of on-state collector-emitter voltage ($V_{ce,on}$) via electro-thermal coupling relationships. To eliminate temperature-dependent parameter drift, a junction temperature ($T_{J}$) compensation algorithm was implemented through calibration at reference current $I_{c,inf}$. Experimental validation demonstrated strong concordance between oscilloscopic measurements and model-derived $V_{ce,on}$ values within ±3.3% error bounds. Comparative analysis revealed a 0.96 correlation coefficient between thermal gradient-based $V_{ce,on}$ estimations and direct thermal impedance measurements. This non-invasive monitoring paradigm achieves simultaneous assessment of wire bond degradation and solder layer fatigue through multivariate thermal signature analysis. The proposed methodology provides three principal advantages: (1) elimination of additional electrical sensors through thermal–electrical analogy modeling; (2) implementation of temperature drift compensation through parametric normalization; (3) capability for in-situ condition monitoring without module disassembly. Field validation data indicate a noticeable improvement in prognostic accuracy compared to conventional monitoring approaches, significantly enhancing operational reliability in power electronic conversion systems. This study demonstrates the feasibility of the proposed monitoring method under controlled laboratory conditions. While the results are promising, practical implementation in transportation systems requires addressing several challenges, including robust sensor installation in high-voltage environments, adaptation to highly dynamic mission profiles, and integration with existing vehicle monitoring systems. Future work will focus on field validation and addressing these implementation challenges.

## Data Availability

Data is contained within the article.
